# Pride-asap: Automatic fragment ion annotation of identified PRIDE spectra^☆^

**DOI:** 10.1016/j.jprot.2013.04.011

**Published:** 2013-12-16

**Authors:** Niels Hulstaert, Florian Reisinger, Jonathan Rameseder, Harald Barsnes, Juan Antonio Vizcaíno, Lennart Martens

**Affiliations:** a Department of Medical Protein Research, VIB, Ghent, Belgium; b Department of Biochemistry, Ghent University, Ghent, Belgium; c EMBL Outstation, European Bioinformatics Institute, Wellcome Trust Genome Campus, Hinxton, Cambridge, UK; d Computational and Systems Biology Initiative, Massachusetts Institute of Technology, Cambridge, MA, USA; e David H. Koch Institute for Integrative Cancer Research at MIT, Cambridge, MA, USA; f Proteomics Unit, Department of Biomedicine, University of Bergen, Norway

**Keywords:** API, application programming interface, asap, Automatic Spectrum Annotation Pipeline, GUI, graphical user interface, PRIDE, PRoteomics IDEntifications (database), PSM, peptide spectrum match, PTM, post-translational modification, Proteomics, Bioinformatics, Mass spectrometry, PRIDE

## Abstract

We present an open source software application and library written in Java that provides a uniform annotation of identified spectra stored in the PRIDE database. Pride-asap can be ran in a command line mode for automated processing of multiple PRIDE experiments, but also has a graphical user interface that allows end users to annotate the spectra in PRIDE experiments and to inspect the results in detail. Pride-asap binaries, source code and additional information can be downloaded from http://pride-asa-pipeline.googlecode.com.This article is part of a Special Issue entitled: Standardization and Quality Control in Proteomics.

## Highlights

• We have built an automatic spectrum annotation pipeline for PRIDE.
• The tool provides both a GUI and a command-line.
• The provided annotations are robust and consistent.
• The tool can be applied easily to thousands of PRIDE experiments.
• Results are available in the GUI, and as text files for downstream analysis.

## Graphical abstract

**Figure:**
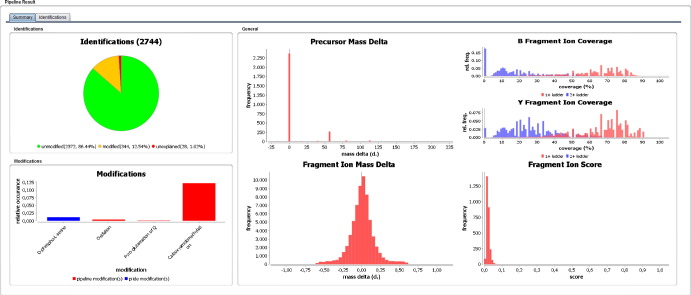

The PRIDE (PRoteomics IDEntifications) database has been collecting proteomics data for several years [1], displaying an exponential growth curve. Over the life span of the PRIDE database, the ability of the system to capture information has increased dramatically, with the addition of (un-)identified mass spectra in 2006 [2] and the storage of fragment ion annotation for identified spectra since 2009 [3]. As a result of these incremental updates, the data stored in PRIDE can vary substantially in the level of annotation provided, both at the level of the peptide and protein identifications, as well as with regard to the experimental meta-information. Even the emergence of tools that aid and standardize data submission, notably the original PRIDE Converter application [4] and the new PRIDE Converter 2 [5], has not been able to fully do away with all existing issues.

One of the areas for improvement is the determination of fragment ion annotation at the peptide-to-spectrum match (PSM) level, which can help researchers to interpret their quality and validity. Indeed, whereas some of the data processing APIs used in PRIDE Converter and PRIDE Converter 2 can determine this annotation based on the output of the search engine (e.g., MascotDatfile [6] and OMSSA Parser [7]), it does not extract such annotation from others (e.g., X!TandemParser [8]). Furthermore, the reported annotation can differ between these different APIs, leading to substantial heterogeneity and thus search engine bias even when annotation is present. As a result, mining PRIDE data for fragmentation characteristics for reuse [9], analysis [10], or quality control [11] is currently a difficult and error-prone enterprise, without any standardization.

In order to alleviate this issue, we here present pride-asap, the automatic spectrum annotation pipeline that provides a homogeneous *a posteriori* fragment ion annotation for PRIDE data, regardless of origin or current annotation status. In contrast to the recent work by Neuhauser et al. [12], pride-asap does not seek to provide the most exhaustive possible annotation for a specific type of high mass accuracy MS/MS spectra, but rather focuses on a rigorous and robust annotation that is compatible with any fragmentation and instrument type, and that will hold across very many independent experiments.

The pipeline uses the PRIDE public MySQL instance that is also used by the PRIDE Inspector [11] as the source data repository. An overview of the entire workflow is provided in Fig. 1. First, for a given experiment, all originally submitted peptide identifications, including any annotated post-translational modifications (PTMs), are loaded. Then a mass recalibration step is performed to determine possible systematic mass errors *per* considered charge state. All identifications with a mass delta *Δm* within a defined window of width 2*ε*, taken to reflect a suitable mass error for the annotated instrument, are taken into account for this recalibration.

$$\left|\mathrm{\Delta m}\right|=\left|m_{e}-m_{t}\right|<\varepsilon$$

The next step in the pipeline attempts to explain each remaining precursor mass deviation larger than *ε* by a combination of possible additional, user-specified post-translational modifications. This step is particularly important for PRIDE experiments submitted before 2008 (PRIDE accession numbers below 9000), where the absence of a standard submission tool often led to errors in the annotation of PTMs. A user-configurable set of commonly encountered modifications is therefore predefined on the pipeline level and can be combined with the modifications found in PRIDE for the given experiment. Modifications with equal mass delta signatures can be handled by the pipeline, but they increase the combinatorial possibilities significantly. After this step, one of three modification states will be assigned to each peptide: (i) unmodified, the precursor mass deviation is smaller than the allowed mass error; (ii) modified, the mass deviation can be explained by a combination of modification masses; or (iii) unexplained: the mass deviation is significant but cannot be explained by any modification combination.

The peptide sequence identifications are then re-matched against their corresponding spectra. An adaptive noise filter based upon iterative winsorization [13] is first applied to each spectrum. This technique calculates a spectrum-specific noise threshold value by iteratively reducing intensity outliers, determined as any intensity outside the window centered on the median with a width equal to twice the median absolute deviation. The remaining ions in the filtered spectrum are subsequently annotated, in turn allowing the peptide-to-spectrum match to be scored. Annotation is performed by matching calculated single and double charged b- and y-ions for the precursor peptide sequence against the spectrum peaks. The average fragment ion score is then defined as

$$s_{\mathrm{avg}}=\frac{\frac{I_{m}}{I_{t}}}{\left|P_{m}\right|}$$

where *I_m_* is the summed intensity of the matching peaks, *I_t_* is the total peak intensity and *|P_m_|* is the number of matched peaks. This score is primarily used to choose the best match for the modified peptides where more than one possible combination or localization of modifications can be constructed for the observed precursor mass deviation.

The final result of the pipeline can be directly visualized in the graphical user interface (GUI, see Fig. 2), but will also be written to two files. The first file contains the fragment ion annotations, scores and spectrum metadata for all identifications in an experiment. This tab-separated text file can later be re-imported for visualization in the GUI or can be loaded into a downstream data analysis program such as a spreadsheet or R [14] for further analysis. The second file is formatted as XML and contains the modifications used to explain the observed precursor mass deviations. This file can also be re-imported into the pipeline GUI to serve as a fixed set of modifications for the annotation of another experiment, if deemed appropriate. The pipeline can be configured in detail through parameters accessible in the GUI, or through a properties file for command line usage. The GUI also provides the user with a concise overview of the resulting annotations and their quality, through summary charts that detail the mass deviations, modifications used and fragment ion coverage statistics (Fig. 2b).

The pride-asap Java application is open source under the permissive Apache2 license. The Spring 3 framework is used for both for querying the PRIDE public MySQL instance as well as for dependency injection, thus making the application easily pluggable; all pipeline components are loosely coupled by means of interfaces and can thus be replaced at will. This is handled dynamically through two XML files, one for the GUI and the other for command line mode, allowing new implementations to be plugged in at load time.

The pipeline has already been used in production in two recent studies [15] and [16], and has proven to be capable of automatically processing more than a thousand PRIDE experiments without issues. The pride-asap pipeline will also allow applications such as PRIDE Inspector to show uniform spectrum annotations across all PRIDE experiments, and to guarantee consistent visualization of protein and peptide identification data loaded from the standard mzIdentML [17] format, where the provision of fragment ion annotation is optional. It will also provide a solid basis on which to build an *a posteriori* quality control framework for the PRIDE database [18,19]. Additionally, the tool has now been included in the latest version of the PRIDE Inspector tool [11] (version 1.3.0) as well, where it can be used to retroactively annotate experiments from within PRIDE Inspector.

N.H. and J.A.V. are funded by the ‘ProteomeXchange’ project grant agreement number 260558, funded by the European Union 7th Framework Program. J.A.V. is also supported by the EU FP7 project ‘LipidomicNet’ [grant agreement number 202272]. F.R. is supported by the Wellcome Trust [grant number WT085949MA]. J.R. is funded by the Howard Hughes Medical Institute International Student Research Fellowship. H.B. is supported by the Research Council of Norway. L.M. acknowledges the support of Ghent University (Multidisciplinary Research Partnership “Bioinformatics: from nucleotides to networks”), and the PRIME-XS project, grant agreement number 262067 funded by the European Union 7th Framework Program.

The authors declare no conflict of interest.

## Figures and Tables

**Fig. 1:**
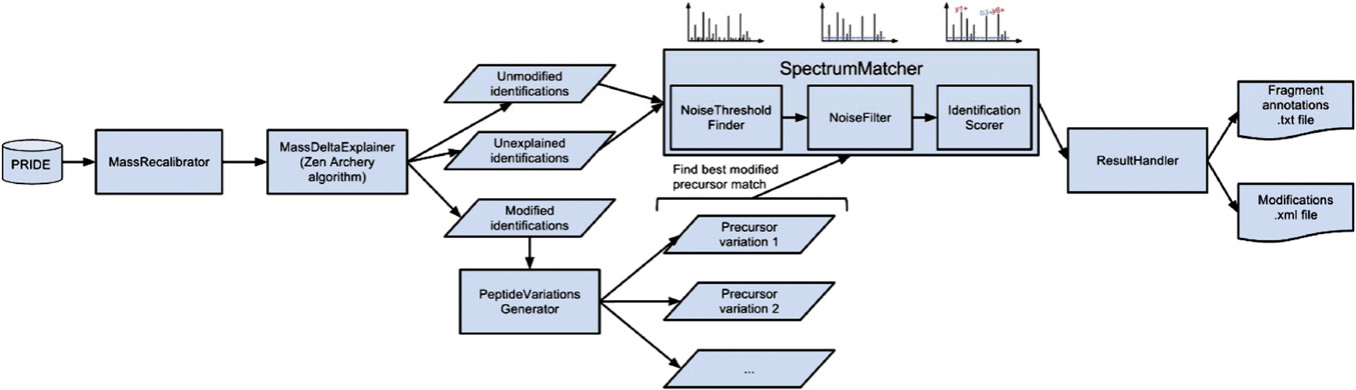
Overview of pride-asap. Identifications and spectra are retrieved from the PRIDE public MySQL database, and processed into three categories: unmodified, modified and unexplained spectra. The peptide sequences are then matched to the corresponding spectra after adaptive noise filtering, and a score is derived for each peptide-to-spectrum match. The final output of the tool consists of the complete list of annotated identifications and spectra, and the list of modifications used to explain the observed precursor mass deviations in that experiment.

**Fig. 2:**
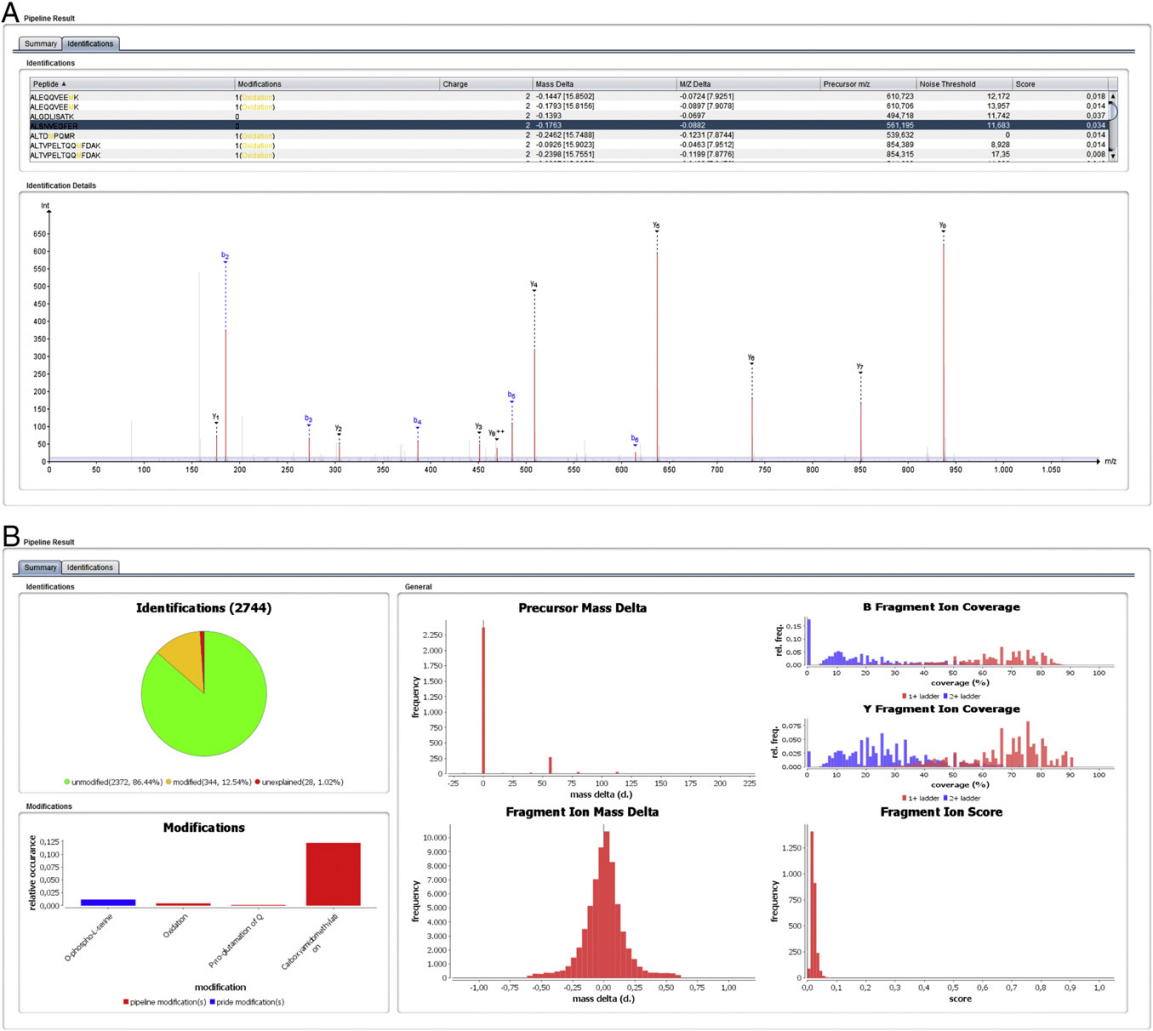
Screenshot of the pride-asap graphical user interface. (a) shows the list of annotated identifications (top) and the annotated spectrum for the currently selected peptide-to-spectrum match. Note the indication of the noise threshold as a blue shaded area. (b) shows the overview charts, that provide summary information on an experiment after annotation, including the distribution of unmodified, modified and unmatched identifications, the mass deltas, b- and y-ion coverages, and the fragment ion score distribution.
